# Low-power phonon lasing through position-modulated Kerr-type nonlinearity

**DOI:** 10.1038/s41598-019-38578-8

**Published:** 2019-02-08

**Authors:** P. Djorwe, Y. Pennec, B. Djafari-Rouhani

**Affiliations:** Institut d’Electronique, de Microélectronique et Nanotechnologie, UMR CNRS 8520 Université de Lille, Sciences et technologies, Villeneuve d’ Ascq, 59652 France

## Abstract

We demonstrate low-power amplification process in cavity optomechanics (COM). This operation is based on the nonlinear position-modulated self-Kerr interaction. Owing to this nonlinear term, the effective coupling highly scales with the photon number, resulting in a giant enhancement of the cooperativity. Even for small nonlinearity, the system reaches the amplification threshold for weak driving strength, leading to low-power phonon lasing. This amplifier can be phase-preserving and provides a practical advantage related to the power consumption issues. This work opens up new avenues to perform low-power and efficient amplifiers in optomechanics and related fields.

## Introduction

Cavity optomechanics (COM), which is devoted to explore interaction between electromagnetic radiation and mechanical object, provides a platform to perform phonon lasing action^1,2^ based on backaction amplification^3^. At the threshold of parametric instability, backaction-induced mechanical gain overcomes mechanical loss, resulting to an amplification process that leads to coherent phonon oscillations^1^. Similar to stimulated emission of photon lasing in cavity with a gain medium, backaction amplification induces stimulated phonon lasing from the parametric instability’s threshold^4^. Both phonon lasing and amplification are actively studied in optomechanics, and other fields, for technological purposes.

Single-phonon Fock state detection has been recently performed in^5,6^, and that paves a way to variety of quantum state engineering tasks including, quantum information processing^7^ and quantum entanglement of remote mechanical elements^8^. The development of single phonon source reveals also a process towards technologies for precision sensing^9^. Moreover, intensive researches are carried out in order to improve these achievements, for instance to lower the power need for phonon lasing. Such performance has been recently realized using ${\mathscr{P}}{\mathscr{T}}$-symmetry optomechanics^10^, and the polarization of light in coupled optomechanical devices^11^.

To use quantum signals, those involving only a few quanta, they need to be amplified and must display some degree of purity to carry certain amount of information. This issue can be handled by characterizing the amplification process as the system gets closer to the phonon lasing threshold. These characteristics include phase-preserving amplification^12–14^, the power gain, the added noise, and the gain-bandwidth product^15–18^. The purpose of a phase-preserving amplifier is to make a weak signal large, regardless of its phase, so that it can be perceived by devices unable to resolve the original signal, while sacrificing as little as possible in signal-to-noise ratio^14^. Engineering amplifier having large power gain with quantum-limited added noise^17,18^, and without limitation on the gain-bandwidth product^17^ are useful for applications such as sensing^3,9^ and quantum information processing^7^. Another interesting feature of amplifiers is their ability to reach high gain amplification for low input power. Such an amplifier has been recently realized, and has shown high gain for low-power operation and low quantum limit noise performance^19^.

Recently, nonlinear position-modulated self-Kerr interaction has been engineered in COM, and it was found that it leads to an effective coupling that scales with the square of the photon number^20^. Such nonlinearity can be derived in the situation where Kerr nonlinear coefficient is modulated by the mechanical position. Cavity polaritons have been recently proposed as good candidates for such interactions in optomechanical systems^21^. Position modulation of Kerr nonlinearity can be possible in optical cavity, where the entire space between the two mirrors (where one of them can move) of the cavity is filled by a *χ*^(3)^ medium. However, the resulting position-dependent Kerr term is weak, since it is inversely proportional to the cavity length^20^. Therefore, the promising systems to implement this kind of interaction are superconducting microwave resonators, where a large optical Kerr nonlinearity at microwave frequencies can be generated^22^. Beside of microwave systems, atom-optical systems can be also used for such nonlinear couplings. Position-dependent Kerr nonlinearity has recently led to a strong coupling, with a magnitude exceeding the strength of the Kerr coefficient, even for low driving strength^20,21^. In the red sideband regime, these authors have demonstrated motional cooling, mode splitting and multistability for low-power^20^. Owing to these interesting nonlinear phenomena, our aim here is to perform low-power phonon laser at the blue sideband, resulting from low-power amplification induced by this nonlinearity. To understand this low-power operation, we have derived the cooperativity that highly scales with the photon number. This pushes the system fastly near to the amplification’s threshold. We have characterized this amplifier, which shows high gain and phase-preserving close to the phonon lasing threshold. This work opens up promising ways to develop low-power amplifiers based on the position-modulated self-Kerr interaction in COMs and superconducting microwave setups^22,23^.

## Results

### Hamiltonian and dynamical equations

Position-modulated self-Kerr nonlinearity can be engineered in COMs^24–26^ or in the superconducting microwave setups exhibiting a giant Kerr nonlinearity^22^. The idea of this engineering is based on the fact that, the Kerr nonlinear coefficient is modulated by the position of the mechanical resonator connected to the system. Such nonlinear interaction, has been recently investigated in^20,21^. This has led to an effective coupling that highly scales with the photon number. In the red sideband, this effective coupling leads to low powers required for motional cooling, the emergence of multistability, and other interesting nonlinear features. Owing to these exciting nonlinear effects, here we move to the blue sideband and investigate on phonon lasing and amplification phenomenon. In the frame rotating at the driving field frequency *ω*_*p*_, the Hamiltonian describing the generic system (with $\hslash =1$) is given by,

1 $$H=-\,{{\rm{\Delta }}}_{0}{a}^{\dagger }a+{\omega }_{m}{b}^{\dagger }b-{g}_{l}{a}^{\dagger }a({b}^{\dagger }+b)-{g}_{nl}{a}^{\dagger }{a}^{\dagger }aa({b}^{\dagger }+b)+E(a+{a}^{\dagger }\mathrm{).}$$

In this Hamiltonian, *a* (*b*) is the annihilation bosonic operator for the intracavity field (mechanical resonator), ${H}_{l,int}=-\,{g}_{l}{a}^{\dagger }a({b}^{\dagger }+b)$ and ${H}_{nl,int}=-\,{g}_{nl}{a}^{\dagger }{a}^{\dagger }aa({b}^{\dagger }+b)$ describe the linear and nonlinear interactions. The first two terms represent the cavity and mechanical free energy respectively, while the last term stands for the driving energy. The parameters *ω*_*m*_ and ${{\rm{\Delta }}}_{0}={\omega }_{p}-{\omega }_{cav}$ are the mechanical frequency of the resonator and the detuning between the driving (*ω*_*p*_) and the cavity eigenfrequency (*ω*_*cav*_). The linear and nonlinear optomechanical couplings are denoted by *g*_*l*_ and *g*_*nl*_, respectively. The mechanical displacement is defined as $x={x}_{ZPF}(b+{b}^{\dagger })$, where ${x}_{ZPF}=\sqrt{\tfrac{\hslash }{2m{\omega }_{m}}}$ is the zero-point fluctuation amplitude of the mechanical resonator, with *m* its effective mass. From the Hamiltonian given in Eq. (1), the Nonlinear Langevin Equations (NLEs), including cavity (*κ*) and mechanical (*γ*_*m*_) dissipations, can be derived as,

2 $$\{\begin{array}{rcl}\dot{a} & = & [i({{\rm{\Delta }}}_{0}+{g}_{l}({b}^{\dagger }+b)+2{g}_{nl}({b}^{\dagger }+b){a}^{\dagger }a)-\frac{\kappa }{2}]a-iE,\\ \dot{b} & = & -(i{\omega }_{m}+\frac{{\gamma }_{m}}{2})b+i{g}_{l}{a}^{\dagger }a+i{g}_{nl}{a}^{\dagger }{a}^{\dagger }aa\mathrm{.}\end{array}$$

Throughout the work, we assume the hierarchy of parameters ${\gamma }_{m},\,{g}_{l}\ll \kappa \ll {\omega }_{m}$, similar to the experiments carried out in the resolved sideband regime^5,6^. Our numerical and analytical investigations will be done at the sideband ${{\rm{\Delta }}}_{0}={\omega }_{m}$. To get insight of the phonon lasing phenomenon, we linearize Eq. (2) in the limit of large driving field. In this case, both intracavity field (*a*) and mechanical degrees of freedom (*b*) can be splitted into their average fields (*α*(*t*), *β*(*t*)) with some amount of fluctuations (*δα*(*t*), *δβ*(*t*)) as follows,

3 $$\{\begin{array}{rcl}\delta \alpha (t) & = & a(t)-\alpha (t),\\ \delta \beta (t) & = & b(t)-\beta (t\mathrm{).}\end{array}$$

Using Eq. (3) in Eq. (2), leads to the steady-state dynamics,

4 $$\{\begin{array}{rcl}\dot{\alpha } & = & (i{\rm{\Delta }}-\frac{\kappa }{2})\alpha +\sqrt{\kappa }{\alpha }^{in},\\ \dot{\beta } & = & -(i{\omega }_{m}+\frac{{\gamma }_{m}}{2})\beta +i{g}_{l}{|\alpha |}^{2}+i{g}_{nl}{|\alpha |}^{4},\end{array}$$

with the corresponding lowest order fluctuations dynamics, including noises,

5 $$\{\begin{array}{rcl}\delta \dot{\alpha } & = & (i{\rm{\Delta }}-\frac{\kappa }{2})\delta \alpha +i\chi (\delta {\beta }^{\ast }+\delta \beta )+i\eta (\delta {\alpha }^{\ast }+\delta \alpha )+\sqrt{\kappa }\delta {\alpha }^{in},\\ \delta \dot{\beta } & = & -(i{\omega }_{m}+\frac{{\gamma }_{m}}{2})\delta \beta +i\chi (\delta {\alpha }^{\ast }+\delta \alpha )+\sqrt{{\gamma }_{m}}\delta {\beta }^{in}.\end{array}$$

In Eq. (4) and Eq. (5), we have set for convenience $-iE=\sqrt{\kappa }{\alpha }^{in}$ ^18^ where the driving strength *α*^*in*^ is related to the input power *P*_*in*_ as ${\alpha }^{in}=\sqrt{\frac{{P}_{in}}{\hslash {\omega }_{p}}}$. We have defined the effective coupling as $\chi ={\chi }_{0}\alpha$ with ${\chi }_{0}=({g}_{l}+2{g}_{nl}|\alpha {|}^{2})$, $\eta =4{g}_{nl}|\alpha {|}^{2}{\rm{Re}}(\beta )$ and ${\rm{\Delta }}={{\rm{\Delta }}}_{0}+2{\chi }_{0}{\rm{Re}}(\beta )$. The noise operators are characterized by the *noncommuting* properties, i.e., $\langle \delta {\alpha }^{in}(t)\rangle =0$, $\langle \delta {\alpha }^{in\dagger }(t^{\prime} )\delta {\alpha }^{in}(t)\rangle ={n}_{\alpha }\delta (t^{\prime} -t)$, and $\langle \delta {\alpha }^{in}(t^{\prime} )\delta {\alpha }^{in\dagger }(t)\rangle =({n}_{\alpha }+\mathrm{1)}\delta (t^{\prime} -t)$ for the input field and $\langle \delta {\beta }^{in}(t)\rangle =0$, $\langle \delta {\beta }^{in\dagger }(t^{\prime} )\delta {\beta }^{in}(t)\rangle ={n}_{th}\delta (t^{\prime} -t)$, and $\langle \delta {\beta }^{in}(t^{\prime} )\delta {\beta }^{in\dagger }(t)\rangle =({n}_{th}+\mathrm{1)}\delta (t^{\prime} -t)$ for the thermal bath. The quantities *n*_*α*_ and *n*_*th*_ are the equilibrium occupation numbers for the input field and mechanical oscillator, respectively.

### Stability

The steady-state solutions *α*_*s*_ and *β*_*s*_ are derived from Eq. (4), providing $\dot{\alpha }=0$ and $\dot{\beta }=0$. These solutions are given by,

6 $$\{\begin{array}{rcl}{\alpha }_{s} & = & \frac{\sqrt{\kappa }{\alpha }^{in}}{(\frac{\kappa }{2}-i[{{\rm{\Delta }}}_{0}+2{\chi }_{0}{\rm{Re}}({\beta }_{{\rm{s}}})])},\\ {\beta }_{s} & = & ({g}_{l}+{g}_{nl}{|{\alpha }_{s}|}^{2})\frac{{|{\alpha }_{s}|}^{2}}{{\omega }_{m}-i\frac{{\gamma }_{m}}{2}}.\end{array}$$

By setting the intracavity intensity as *I* = |*α*_*s*_|^2^, one shows from Eq. (6) that it is solution of the following seventh-order polynomial equation,

7 $${I}^{7}+{a}_{6}{I}^{6}+{a}_{5}{I}^{5}+{a}_{4}{I}^{4}+{a}_{3}{I}^{3}+{a}_{2}{I}^{2}+{a}_{1}I+{a}_{0}=\mathrm{0,}$$

with ${a}_{6}=\frac{3{g}_{l}}{{g}_{nl}}$; ${a}_{5}=\frac{13{g}_{l}^{2}}{4{g}_{nl}^{2}}$; ${a}_{4}=\frac{3{g}_{l}^{3}+{\rm{\Delta }}{\omega }_{m}{g}_{nl}}{2{g}_{nl}^{3}}$; ${a}_{3}=\frac{{g}_{l}({g}_{l}^{3}+3{\rm{\Delta }}{\omega }_{m}{g}_{nl})}{4{g}_{nl}^{4}}$; ${a}_{2}=\frac{{\rm{\Delta }}{\omega }_{m}{g}_{l}^{2}}{4{g}_{nl}^{4}}$; ${a}_{1}=\frac{(\frac{{\kappa }^{2}}{4}+{{\rm{\Delta }}}^{2}){\omega }_{m}^{2}}{{\mathrm{(2}{g}_{nl})}^{4}}$; ${a}_{0}=-\,\frac{\kappa {({\alpha }^{in}{\omega }_{m})}^{2}}{{\mathrm{(2}{g}_{nl})}^{4}}$. These steady state solutions are physically meaningless, unless they are stable. The stability can be given explicitly through Routh-Hurwitz criterion^26^. However, we analyze it here through linear stability theory, and confirm it with parametric instability threshold, since we are in the blue sideband regime. To this end, we start by writing Eq. (5) in the follwing compact form,

8 $$\delta \dot{X}=M\delta X+\varepsilon ,$$

with $\delta X={(\delta \beta ,\delta {\beta }^{\ast },\delta \alpha ,\delta {\alpha }^{\ast })}^{T}$ and $\varepsilon ={(\sqrt{{\gamma }_{m}}\delta {\beta }^{in},\sqrt{{\gamma }_{m}}\delta {\beta }^{in\ast },\sqrt{\kappa }\delta {\alpha }^{in},\sqrt{\kappa }\delta {\alpha }^{in\ast })}^{T}$. The matrix *M* is given by,

9 $$M=[\begin{array}{cccc}-(i{\omega }_{m}+\frac{{\gamma }_{m}}{2}) & 0 & i\chi  & i\chi \\ 0 & (i{\omega }_{m}-\frac{{\gamma }_{m}}{2}) & -i\chi  & -i\chi \\ i\chi  & i\chi  & (i\tilde{{\rm{\Delta }}}-\frac{\kappa }{2}) & i\eta \\ -i\chi  & -i\chi  & -i\eta  & -(i\tilde{\Delta }+\frac{\kappa }{2})\end{array}],$$

with $\tilde{{\rm{\Delta }}}={\rm{\Delta }}+\eta$, and where we have chosen the phase reference of the cavity field so that *α*_*s*_ is real.

**Figure 1:**
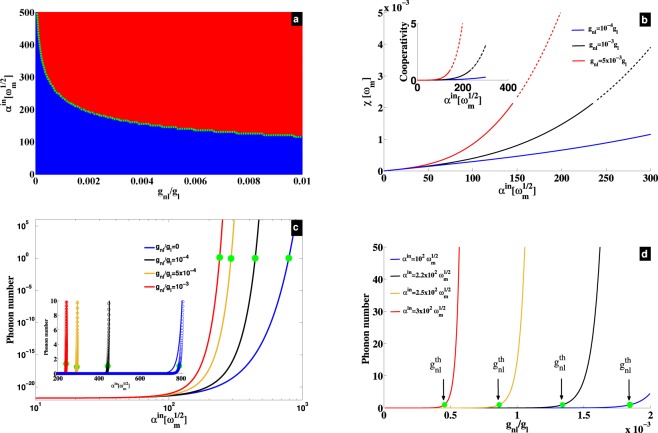
(**a**) Stability diagram. Blue (red) color is stable (unstable). The green dashed curve shows the border between stable and unstable regions, and corresponds to the lasing threshold ${\mathscr{C}}=1$, with the cooperativity ${\mathscr{C}}=4{\chi }^{2}/({\gamma }_{m}\kappa )$. (**b**) Effective coupling *χ* versus *α*^*in*^ for different values of *g*_*nl*_. Full (dashed) curves are stable (unstable). The inset shows the cooperativity ${\mathscr{C}}$ versus *α*^*in*^, for the corresponding values of *g*_*nl*_. (**c**) Phonon number versus *α*^*in*^ in log-log scale, showing the fast increase near the thresholds. The inset displays the same figure in the linear scale, where full curves are from numerical simulation of classical version of Eq. (2), while the dotted lines represent the analytical approximation from $N \sim \exp (-\,{\gamma }_{{\rm{eff}}}t)$. (**d**) Analytical (approximated) phonon number versus *g*_*nl*_. In (**c**,**d**), the different lasing thresholds (${\mathscr{C}}=1$) are indicated by the green dots, namely ${\alpha }_{th}^{in}$ and ${g}_{nl}^{th}$ for the input field and the nonlinear coefficient, respectively. The used parameters are^20,23^, ${\gamma }_{m}={10}^{-3}{\omega }_{m}$, $\kappa =2\times {10}^{-2}{\omega }_{m}$, ${g}_{l}=2\times {10}^{-5}{\omega }_{m}$, and ${{\rm{\Delta }}}_{0}={\omega }_{m}$.

The system is stable if all the real part of eigenvalues (${\lambda }_{i=\mathrm{1..4}}$) of the matrix *M* are negative (${\rm{Re}}({\lambda }_{i=\mathrm{1..4}}) < 0$). This stability depends on steady-state solutions *α*_*s*_ and *β*_*s*_, and is shown in Fig. 1a. The blue color in Fig. 1a depicts stable parameters space, while the red area shows the unstable zone. As the self-Kerr term (*g*_*nl*_) is increasing, the system becomes unstable and the stability is limited for relatively weak driving strength *α*^*in*^. The green dashed line in Fig. 1a holds for the condition ${\mathscr{C}}=1$, where ${\mathscr{C}}=4{\chi }^{2}/({\gamma }_{m}\kappa )={\gamma }_{opt}/{\gamma }_{m}$ generalizes the cooperativity at the limit ${g}_{nl}\ne 0$. This cooperativity, with *γ*_*opt*_ being the optical damping, depicts the border between stable (linear) and unstable (nonlinear) regimes. Moreover, ${\mathscr{C}}=1$ defines the threshold of the phonon lasing in the optomechanical blue sideband. It results that the self-Kerr nonlinearity induces low power phonon lasing action, that can be understood by the enhancement of the effective coupling (*χ*) shown in Fig. 1b. This coupling enhancement is a direct consequence of the fact that *χ* highly scales with the photon number in the presence of *g*_*nl*_, leading to a large cooperativity (see inset of Fig. 1b). Hence, as *g*_*nl*_ increases, the lasing threshold is shifted towards weak driving strength *α*^*in*^, revealing low-power phonon lasing in our proposal.

### Low power phonon lasing

To evaluate the stimulated emission phonon number, we have simulated the classical equivalent of the nonlinear equation given in Eq. (2). This is valid for large enough photon number in the system ($|\alpha {|}^{2}\gg 1$), so that both fluctuations from intracavity field and mechanical resonator can be neglected. By Fast Fourier Transforming (FFT) the results, and collecting the mechanical peaks at the resonance, we can obtain the phonon number versus the driving field *α*^*in*^. Analytical results can be also obtained through an approximated analysis that is detailed in section Methods. Figure 1c displays a log-log scale representation, and it shows that the effect of the nonlinear term *g*_*nl*_ is negligible on the phonon number in the linear regime (${\alpha }^{in}\lesssim {10}^{2}\sqrt{{\omega }_{m}}$). However, this nonlinearity strongly enhances the increase of the phonon number near the instability thresholds. The linear scale representation of the phonon number is displayed in the inset of Fig. 1c, where a comparison is made with the full nonlinear results. Indeed, full and dotted lines show the numerical and analytical results, respectively. This yields good agreement between numerical calculation and analytics, that is detailed in section Methods. The input fields at the lasing threshold (${\alpha }_{th}^{in}$) are depicted by the green dots and can be obtained through the condition *γ* = *γ*_*opt*_ (or ${\mathscr{C}}=1$). As *g*_*nl*_ is increasing, Fig. 1c reveals a low driving strength *α*^*in*^ required for phonon lasing threshold. In Fig. 1d, we have represented the phonon number versus *g*_*nl*_ for different *α*^*in*^. It results that *g*_*nl*_ enhances stimulated emission of phonons. Indeed, for ${g}_{nl} \sim 0$, there is no lasing up to ${\alpha }^{in}=3\times {10}^{2}\sqrt{{\omega }_{m}}$ in Fig. 1d. Therefore, by adding a small amount of nonlinearity to the system, the lasing threshold rises up and is shifted towards small *g*_*nl*_ as *α*^*in*^ increases. Briefly speaking, the higher is the driving strength, smaller is the amount of nonlinearity (*g*_*nl*_) to reach the lasing threshold, and vice-versa. The nonlinear coefficients required to reach the lasing threshold are indicated by the green dots, namely ${g}_{nl}^{th}$. This can be understood from the dynamics of *b*(*t*) in Eq. (2), showing that *g*_*nl*_ supplies energy (∝*g*_*nl*_|*α*|^4^) to drive the mechanical resonator. This reveals why self-Kerr nonlinearity studied here, is quite interesting and different from Kerr nonlinearity^22,24,26^, quadratic nonlinearity^25,27^ and Duffing nonlinearities^28^ also studied in optomechanics. Indeed, these nonlinearities either shift the cavity frequency or the mechanical frequency, but none of them is directly driving the mechanical resonator. Free-carrier and thermal nonlinearities can compete with self-Kerr nonlinearity, but their effects are negligible in the weak driving limit considered in our work.

### Low power amplification

As self-Kerr nonlinearity enhances low-power phonon lasing (Fig. 1c), this also reveals low-power amplification process in the system. The feature of the nondegenerate parametric amplifier here is to convert a pump mode photon into, one photon signal mode and one idler phonon mode. This can lead to the fact that weak incident signals are amplified, with a minimum possible added noise. Such amplification process can be seen from the linearization of the interaction Hamiltonian of our system. Indeed, the interaction of our system is captured by the Hamiltonian,

10 $${H}_{int}={H}_{l,int}+{H}_{nl,int}=-\,{g}_{l}{a}^{\dagger }a({b}^{\dagger }+b)-{g}_{nl}{a}^{\dagger }{a}^{\dagger }aa({b}^{\dagger }+b),$$

which leads to its linearized form,

11 $${H}_{int}^{lin}=-\,\chi (\delta {\alpha }^{\dagger }\delta {\beta }^{\dagger }+\delta \alpha \delta \beta )-\chi (\delta {\alpha }^{\dagger }\delta \beta +\delta \alpha \delta {\beta }^{\dagger }),$$

after having used Eq. (3). Furthermore, Eq. (11) has been obtained by omitting static terms since they are taken into account in the frequency shift $\tilde{{\rm{\Delta }}}$, and the higher order fluctuations terms have been neglected as being smaller than *α*_*s*_. The second term on the right-hand side of Eq. (11) stands for the counter rotating terms ${H}_{CR}=-\,\chi (\delta {\alpha }^{\dagger }\delta \beta +\delta \alpha \delta {\beta }^{\dagger })$ and can be neglected in the rotating wave approximation (RWA)^1^. However, the first term $({H}_{R}=-\,\chi (\delta {\alpha }^{\dagger }\delta {\beta }^{\dagger }+\delta \alpha \delta \beta ))$ describes a nondegenerate parametric amplifier, where a pump mode (photon) is converted into two quanta, one in the signal mode (photon), and the other in the idler (phonon). To characterize this amplifier, we neglect non resonant terms in Eq. (5) and rewrite it in the RWA as,

12 $$\{\begin{array}{rcl}\delta \dot{\alpha } & = & (i{\rm{\Delta }}-\frac{\kappa }{2})\delta \alpha +i\chi \delta {\beta }^{\dagger }+\sqrt{\kappa }\delta {\alpha }^{in},\\ \dot{\delta }{\beta }^{\dagger } & = & (i{\omega }_{m}-\frac{{\gamma }_{m}}{2})\delta {\beta }^{\dagger }-i\chi \delta \alpha +\sqrt{{\gamma }_{m}}\delta {\beta }^{in}.\end{array}$$

By solving Eq. (12) in the Fourier space, together with the input-output relation, $\delta {\alpha }^{out}=\delta {\alpha }^{in}-\sqrt{\kappa }\delta \alpha$ ^15,18^, we can evaluate the output field *δα*^*out*^. This output field is a key element to characterize the gain and added noise of the amplifier. More details on calculations leading to the gain and added noise are reported in section Methods, while specific results are shown in what follows. The input-output relation leads to the output field,

13 $$\delta {\alpha }^{out}=(1-\sqrt{\kappa }{\chi }_{eff}^{c})\delta {\alpha }^{in}-i\sqrt{\kappa }{\eta }_{c}\delta {\beta }^{in\dagger },$$

where

14 $${\eta }_{c}=\frac{{\chi }_{m}{\chi }_{c}\chi \sqrt{{\gamma }_{m}}}{1-{\chi }_{m}{\chi }_{c}{\chi }^{2}};\,{\chi }_{eff}^{c}=\frac{{\chi }_{c}\sqrt{\kappa }}{1-{\chi }_{m}{\chi }_{c}{\chi }^{2}},$$

with the susceptibilities ${\chi }_{c}={[\frac{\kappa }{2}-i(\omega +\tilde{{\rm{\Delta }}})]}^{-1}$, and ${\chi }_{m}={[\frac{{\gamma }_{m}}{2}-i(\omega +{\omega }_{m})]}^{-1}$. In Eq. (13), the coefficient in front of the incident signal *δα*^*in*^ characterizes the amplification gain, while the one in front of the thermal noise informs on the added noise. These characteristics are deduced from the output noise power spectral density (PSD) defined as^17,18^,

15 $${S}_{out}=\frac{1}{2}(\langle \delta {\alpha }^{out\dagger }\delta {\alpha }^{out}+\delta {\alpha }^{out}\delta {\alpha }^{out\dagger }\rangle ).$$

**Figure 2:**
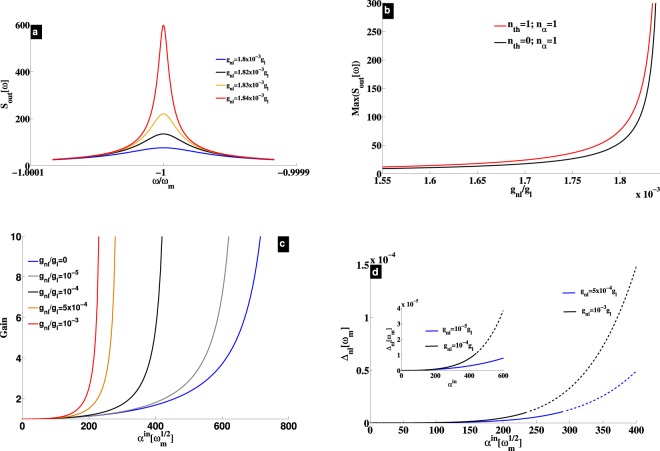
(**a**) Output noise power spectral density (PSD) for different values of *g*_*nl*_. The magnitude of ${S}_{out}=\sqrt{\langle \delta {\alpha }^{out\dagger }\delta {\alpha }^{out}\rangle }$ reveals the amplitude of the mechanical resonator, detected at the output for a given frequency. (**b**) Values of the output spectrum (in (**a**)) at the resonance ($\omega =-\,{\rm{\Delta }}$). (**c**) Amplifier’s gain (see Eq. (16)) versus *α*^*in*^, for different values of *g*_*nl*_. (**d**) Cavity frequency shift ${{\rm{\Delta }}}_{nl}=\tilde{{\rm{\Delta }}}-{{\rm{\Delta }}}_{0}$ versus *α*^*in*^. The driving strength in (**a**,**b**) is ${\alpha }^{in}=2\times {10}^{2}\sqrt{{\omega }_{m}}$, with ${n}_{th}={n}_{\alpha }=1$ for (**a**). The other parameters are the same as in Fig. 1.

The output PSD (*S*_*out*_) is shown in Fig. 2a, and reveals an amplification process at the resonance $\omega =-\,{\rm{\Delta }}$, induced by the nonlinear term *g*_*nl*_. For ${\alpha }^{in}=2\times {10}^{2}\sqrt{{\omega }_{m}}$ and ${g}_{nl}\le 1\times {10}^{-3}{g}_{l}$, there is no amplification as shown in Fig. 1c. However, for ${g}_{nl}\approx 1.85\times {10}^{-3}{g}_{l}$, Fig. 2a clearly shows amplification, meaning that *g*_*nl*_ brings the system near the lasing threshold even for a weak driving strength. In order to appreciate this amplification, the output PSD has been plotted at the resonance $\omega =-{\rm{\Delta }}$ (for ${\alpha }^{in}=2\times {10}^{2}\sqrt{{\omega }_{m}}$) versus *g*_*nl*_, and shown in Fig. 2b. The red and black curves correspond to (*n*_*α*_ = 1, *n*_*th*_ = 1) and (*n*_*α*_ = 1, *n*_*th*_ = 0), respectively. It results that thermal noise has an impact on the amplification, revealing the effect of the added noise on the amplifier. This can be pointed out by evaluating both the power gain ${\mathscr{G}}(\omega )$ and the added noise ${\mathscr{N}}(\omega )$. The amplification of the signal *δα*^*in*^ is measured through the gain $\mathrm{|1}-\sqrt{\kappa }{\chi }_{eff}^{c}{|}^{2}$, which can be simplified (see section Methods) at the resonance ($\omega =-\,{\rm{\Delta }}$) as,

16 $${\mathscr{G}}={|\frac{{\mathscr{C}}+1}{1-{\mathscr{C}}}|}^{2}.$$

The noise performance of the amplifier is figured out through the input-referred added noise, defined as ${\mathscr{N}}(\omega )=({S}_{out}-{S}_{in})/{\mathscr{G}}$, where ${S}_{in}=\frac{1}{2}$ is the vacuum input noise driving the cavity. As shown in section Methods, this expression reduces at the resonance to,

17 $${\mathscr{N}}=\frac{4{\mathscr{C}}({n}_{th}+\frac{1}{2})}{{|{\mathscr{C}}+1|}^{2}}+{n}_{\alpha }.$$

It can be seen from Eq. (16) that the gain is greatly enhanced as the system approaches the lasing threshold at the cooperativity ${\mathscr{C}}\to 1$, revealing the amplification process. As ${\mathscr{C}}$ strongly scales with the self-Kerr term *g*_*nl*_, it results an enhancement of amplification induced by *g*_*nl*_ as shown in Fig. 2c. Furthermore, we have ${\mathscr{N}}\to ({n}_{eff}+\frac{1}{2})$ for ${\mathscr{C}}\to 1$, where ${n}_{eff}={n}_{th}+{n}_{\alpha }$ is the effective phonon number of the mechanical resonator. This reveals that the amplifier reaches the quantum limit for phase-preserving^13,14^ near the lasing threshold for *n*_*eff*_ = 0, and if there are no additional loss channels. However, for non zero effective (thermal) phonon number, ${\mathscr{N}}$ linearly increases with *n*_*eff*_, degrading the signal amplification purity as depicted by the red curve in Fig. 2b. This is not the case in the amplifier studied in^13,17^ where the gain can take arbitrarily large values, and where any thermal noise contribution is suppressed for a large-gain limit^17^. Despite of this, the practical advantage of our amplifier is its low power consumption due to the presence of *g*_*nl*_.

## Discussion

We have carried out investigation on position-modulated Kerr-type nonlinearity in blue sideband optomechanics. This is mainly focused on phonon lasing and amplification process. The amplification, which is based on the recorded output field, is shown to be as a manifestation of phonon lasing inside the cavity. Both phenomena are enhanced by this nonlinear term, through the condition ${\mathscr{C}}\to 1$. Indeed, a cooperativity equal to unity fulfills phonon lasing threshold requirement, large gain amplification, and condition of quantum limit for a phase-preserving amplifier. We have shown that these features happen for low-power strength, which is the figure of merit of the used nonlinear term, compared to those known so far^22,24–28^. This is shown in Fig. 1a, where for ${g}_{nl}\ne 0$, the system reaches nonlinear regime for weak driving. Owing to such feature, we were able to drop counter rotating terms in our analytical treatment, since they start to manifest in strong driving limit. The green dashed line in Fig. 1a shows the threshold of phonon lasing using the rotating wave approximation, while the border between stable (blue) and unstable (red) regimes shows the same threshold when counter rotating terms are included. It results a good agreement between numerical and analytical calculations, confirming the validity of the rotating wave approximation in our investigation. Moreover, this is highlighted in Fig. 1c where a good matching between numerical (full curves) and analytical (dashed curves) results is induced by *g*_*nl*_. For acoustic excitations, using weak driving strength to reach phonon lasing and large gain for the amplified phonon source having minimum added noise could be an interesting achievement. This can be useful for phonon information processing, and the position-modulated Kerr-type nonlinearity can be helpful. At the resonance and for ${n}_{eff}\to 0$, the amplifier reaches the quantum limit ${\mathscr{N}}\to \frac{1}{2}$ at the threshold ${\mathscr{C}}\to 1$ no matter is the strength of *g*_*nl*_. For ${n}_{eff}\ne 0$ however, the performance of the amplifier is impaired by both thermal and input field fluctuations as shown in Fig. 2b. Close to the quantum limit and for lower sideband regime, this amplifier performs better when the modified Kerr term is involved. This is because the effective quantum population *n*_*eff*_ is more reduced for ${g}_{nl}\ne 0$ as recently shown in^20^. Moreover, a practical benefit of the amplifier discussed here is its low-power consumption to reach high amplification gain (phonon lasing).

## Methods

Figure 2d shows that the cavity frequency shift ($\tilde{{\rm{\Delta }}}-{{\rm{\Delta }}}_{0}$) is weak in the linear regime that the intracavity field remains at the mechanical sideband $\tilde{{\rm{\Delta }}} \sim {\omega }_{m}$. This allows us to get analytical expression of stimulated phonon number by introducing the slowly varying amplitude variables^29^, $\delta \tilde{\alpha }=\delta \alpha \exp (\frac{\kappa }{2}-i\tilde{{\rm{\Delta }}})t$ and $\delta \tilde{\beta }=\delta \beta \exp (i{\omega }_{m})t$. Using these variables in the cavity field equation in Eq. (12) yields,

18 $$\delta \tilde{\alpha }={\int }_{-\infty }^{t}\,i\chi \delta {\tilde{\beta }}^{\dagger }{e}^{\frac{\kappa }{2}\tau }d\tau .$$

As we are dealing with weak coupling regime in our analysis ($\kappa \gg \chi$, see Fig. 1b), we can adiabatically eliminate *δα*(*t*), then *χ* can be taken out of the integral Eq. (18). Moreover, $\kappa \gg \gamma$ indicates that the evolution of $\delta \tilde{\beta }(t)$ is much slower that *δα*(*t*), meaning that $\delta \tilde{\beta }(t)$ can be considered as a constant term in Eq. (18). Under these conditions, the integration of Eq. (18) yieds,

19 $$\delta \alpha =\frac{2i\chi }{\kappa }\delta {\beta }^{\dagger }.$$

By replacing Eq. (19) in Eq. (12), one gets

20 $$\delta {\dot{\beta }}^{\dagger }=(i{\omega }_{m}-\frac{{\gamma }_{eff}}{2})\delta {\beta }^{\dagger },$$

where the effective damping is ${\gamma }_{eff}={\gamma }_{m}-{\gamma }_{opt}$, with the optical damping ${\gamma }_{opt}=\frac{4{\chi }^{2}}{\kappa }$. The solution of ${\mathscr{C}}=1$ (or ${\gamma }_{eff}=0$), with the cooperativity ${\mathscr{C}}=4{\chi }^{2}/({\gamma }_{m}\kappa )$, gives the lasing threshold shown by the green dashed curve in Fig. 1a. The stimulated phonon number depicted in Fig. 1(c,d), is deduced from Eq. (20), by evaluating $N=\langle \delta {\beta }^{\dagger }\delta \beta \rangle ={N}_{0}{e}^{-{\gamma }_{eff}t}$, with *N*_0_ = 1 the phonon number at *t* = 0^30^.

The amplification shown in Fig. 2(a,b) are obtained by solving Eq. (12) in the frequency domain, together with the input-output relation^15,18^. In the Fourier space, Eq. (12) leads to,

21 $$\{\begin{array}{rcl}-i\omega \delta \alpha  & = & (i\tilde{{\rm{\Delta }}}-\frac{\kappa }{2})\delta \alpha +i\chi \delta {\beta }^{\dagger }+\sqrt{\kappa }\delta {\alpha }^{in},\\ -i\omega \delta {\beta }^{\dagger } & = & (i{\omega }_{m}-\frac{{\gamma }_{m}}{2})\delta {\beta }^{\dagger }-i\chi \delta \alpha +\sqrt{{\gamma }_{m}}\delta {\beta }^{in\dagger }\mathrm{.}\end{array}$$

After some calculations, one obtains

22 $$\{\begin{array}{rcl}\delta \alpha (\omega ) & = & {\chi }_{eff}^{c}\delta {\alpha }^{in}+i{\eta }_{c}\delta {\beta }^{in\dagger },\\ \delta {\beta }^{\dagger }(\omega ) & = & {\eta }_{m}\delta {\beta }^{in\dagger }-i{\chi }_{eff}^{m}\delta {\alpha }^{in},\end{array}$$

where

23 $$\{\begin{array}{llll}{\eta }_{c} & = & \frac{{\chi }_{m}{\chi }_{c}\chi \sqrt{{\gamma }_{m}}}{1-{\chi }_{m}{\chi }_{c}{\chi }^{2}}; & {\chi }_{eff}^{c}=\frac{{\chi }_{c}\sqrt{\kappa }}{1-{\chi }_{m}{\chi }_{c}{\chi }^{2}},\\ {\eta }_{m} & = & \frac{{\chi }_{m}\sqrt{{\gamma }_{m}}}{1-{\chi }_{m}{\chi }_{c}{\chi }^{2}}; & {\chi }_{eff}^{m}=\frac{{\chi }_{m}{\chi }_{c}\chi \sqrt{\kappa }}{1-{\chi }_{m}{\chi }_{c}{\chi }^{2}},\end{array}$$

with the susceptibilities ${\chi }_{c}={[\frac{\kappa }{2}-i(\omega +\tilde{{\rm{\Delta }}})]}^{-1}$, and ${\chi }_{m}={[\frac{{\gamma }_{m}}{2}-i(\omega +{\omega }_{m})]}^{-1}$.

Using the input-output relation, one gets the output field,

24 $$\delta {\alpha }^{out}\mathrm{=(1}-\sqrt{\kappa }{\chi }_{eff}^{c})\delta {\alpha }^{in}-i\sqrt{\kappa }{\eta }_{c}\delta {\beta }^{in\dagger },$$

which leads to the output PSD,

25 $$\begin{array}{rcl}{S}_{out} & = & \frac{1}{2}(\langle \delta {\alpha }^{out\dagger }\delta {\alpha }^{out}+\delta {\alpha }^{out}\delta {\alpha }^{out\dagger }\rangle )\\  & = & \kappa |{\eta }_{c}{|}^{2}({n}_{th}+\frac{1}{2})+\mathrm{|1}-\sqrt{\kappa }{\chi }_{eff}^{c}{|}^{2}({n}_{\alpha }+\frac{1}{2})\\  & = & \kappa |{\eta }_{c}{|}^{2}({n}_{th}+\frac{1}{2})+{\mathscr{G}}({n}_{\alpha }+\frac{1}{2}).\end{array}$$

The amplifier is then characterized by the power gain,

26 $${\mathscr{G}}(\omega )={|1-\sqrt{\kappa }{\chi }_{eff}^{c}|}^{2}={|\frac{1-{{\mathscr{C}}}_{\omega }-2(1-\frac{2i}{{\gamma }_{m}}(\omega +{\omega }_{m}))}{1-{{\mathscr{C}}}_{\omega }}|}^{2},$$

with ${{\mathscr{C}}}_{\omega }=\frac{4}{{\gamma }_{m}\kappa }({\chi }^{2}+(\omega +{\omega }_{m})(\omega +\tilde{{\rm{\Delta }}}))-\frac{2i}{{\gamma }_{m}\kappa }({\gamma }_{m}(\omega +\tilde{{\rm{\Delta }}})+\kappa (\omega +{\omega }_{m}))$. The input-referred added noise quanta to the amplifier is defined as,

27 $$\begin{array}{rcl}{\mathscr{N}}(\omega ) & = & ({S}_{out}-{S}_{in})/{\mathscr{G}}\\  & = & \frac{\kappa |{\eta }_{c}{|}^{2}}{{\mathscr{G}}}({n}_{th}+\frac{1}{2})+{n}_{\alpha }\\  & = & \frac{4{{\mathscr{C}}}_{\omega }({n}_{th}+\frac{1}{2})}{{|1-{{\mathscr{C}}}_{\omega }-2(1-\frac{2i}{{\gamma }_{m}}(\omega +{\omega }_{m}))|}^{2}}+{n}_{\alpha },\end{array}$$

where *S*_*out*_ is given in Eq. (15), and ${S}_{in}=\frac{1}{2}$ holds for the vacuum input noise driving the cavity. At the resonance ($\omega =-\,{\rm{\Delta }}$), these amplifier’s characteristic reduce to,

28 $${\mathscr{G}}={|\frac{{\mathscr{C}}+1}{1-{\mathscr{C}}}|}^{2},$$

and

29 $${\mathscr{N}}=\frac{4{\mathscr{C}}({n}_{th}+\frac{1}{2})}{{|{\mathscr{C}}+1|}^{2}}+{n}_{\alpha },$$

which clearly show: (i) amplification near the lasing threshold (${\mathscr{G}}\to \infty$ for ${\mathscr{C}}\to 1$), and the fact that (ii) the amplifier reaches the quantum limit for a phase-preserving amplifier (${\mathscr{N}}\to \frac{1}{2}$) for ${\mathscr{C}}\to 1$ and ${n}_{eff}\to 0$, with ${n}_{eff}={n}_{th}+{n}_{\alpha }$.
